# 2-[(2,5-Di­methyl­phen­yl)amino]­quinoline-3-carb­oxy­lic acid

**DOI:** 10.1107/S2414314626005468

**Published:** 2026-05-29

**Authors:** Xiao Ji, Sihui Long

**Affiliations:** a School of Chemical Engineering and Pharmacy Wuhan Institute of Technology,Wuhan Hubei 430205 People’s Republic of China; University of Antofagasta, Chile

**Keywords:** hydrogen bond, acid-acid dimer, single crystal

## Abstract

The title compound, C_18_H_16_N_2_O_2_, was synthesized *via* a two-step route with the Buchwald–Hartwig cross-coupling reaction. The quinoline ring system and phenyl ring of the mol­ecule are nearly coplanar with a dihedral angle of 6.51 (5)°. In the crystal, adjacent mol­ecules form carb­oxy­lic acid dimers *via* inter­molecular hydrogen bonding.

## Abstract 2

The title compound was synthesized using a Buchwald–Hartwig cross-coupling reaction followed by hydrolysis. Single crystals were obtained by slow evaporation of an ethanol solution of it at room temperature. In the crystal, adjacent mol­ecules form carb­oxy­lic acid dimers *via* inter­molecular hydrogen bonding.

## Graphical abstract

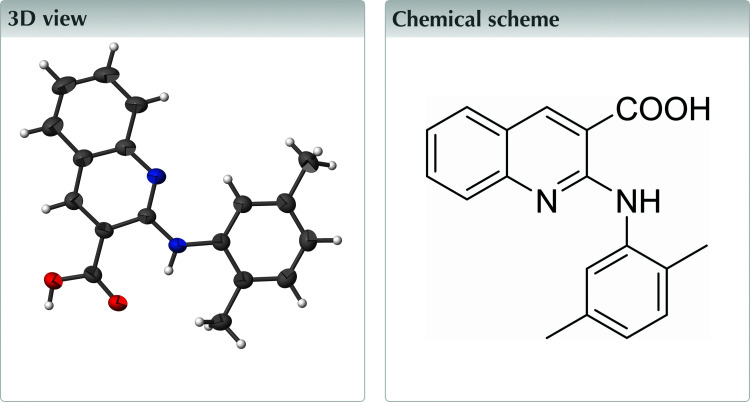

## Structure description

Nonsteroidal anti-inflammatory drugs (NSAIDs) are a class of medicines that exert anti­pyretic, analgesic, and anti-inflammatory effects by inhibiting cyclo­oxygenase (COX). They are widely used in the treatment of rheumatoid arthritis, osteoarthritis, and acute pain (Vishwakarma & Negi, 2020 ▸). Classic NSAIDs such as ibuprofen, naproxen, and flurbiprofen all contain an aryl­propionic acid or aryl­acetic acid structure, and their efficacy is closely related to the carboxyl group in the mol­ecule (Astrvatham *et al.*, 2019 ▸). However, these drugs usually suffer from poor water solubility, variable bioavailability, and gastrointestinal adverse effects. In recent years, studies have shown that the polymorphism of solid drugs directly affects their solubility, dissolution rate, stability, and even biological activity (Bindu *et al.*, 2020 ▸). For example, different polymorphs of ibuprofen exhibit significantly different dissolution behaviors, which in turn affect *in vivo* absorption (Zhou *et al.*, 2024 ▸). Therefore, systematic studies on the polymorphism of NSAIDs are of great significance for optimizing formulation processes, improving therapeutic efficacy, and circumventing patents (Ley *et al.*, 2025 ▸). Research on drug polymorphism holds promise for discovering new crystal forms of drugs, thereby enhancing their druggability and providing a solid scientific basis for generic drug development. Furthermore, clarifying the polymorphic behavior of inter­mediates or products can provide key guidance for subsequent formulation screening, helping to select the thermodynamically stable crystal form with the best bioavailability, thereby reducing the risk of efficacy fluctuations caused by crystal form transformation.

**Figure 1:**
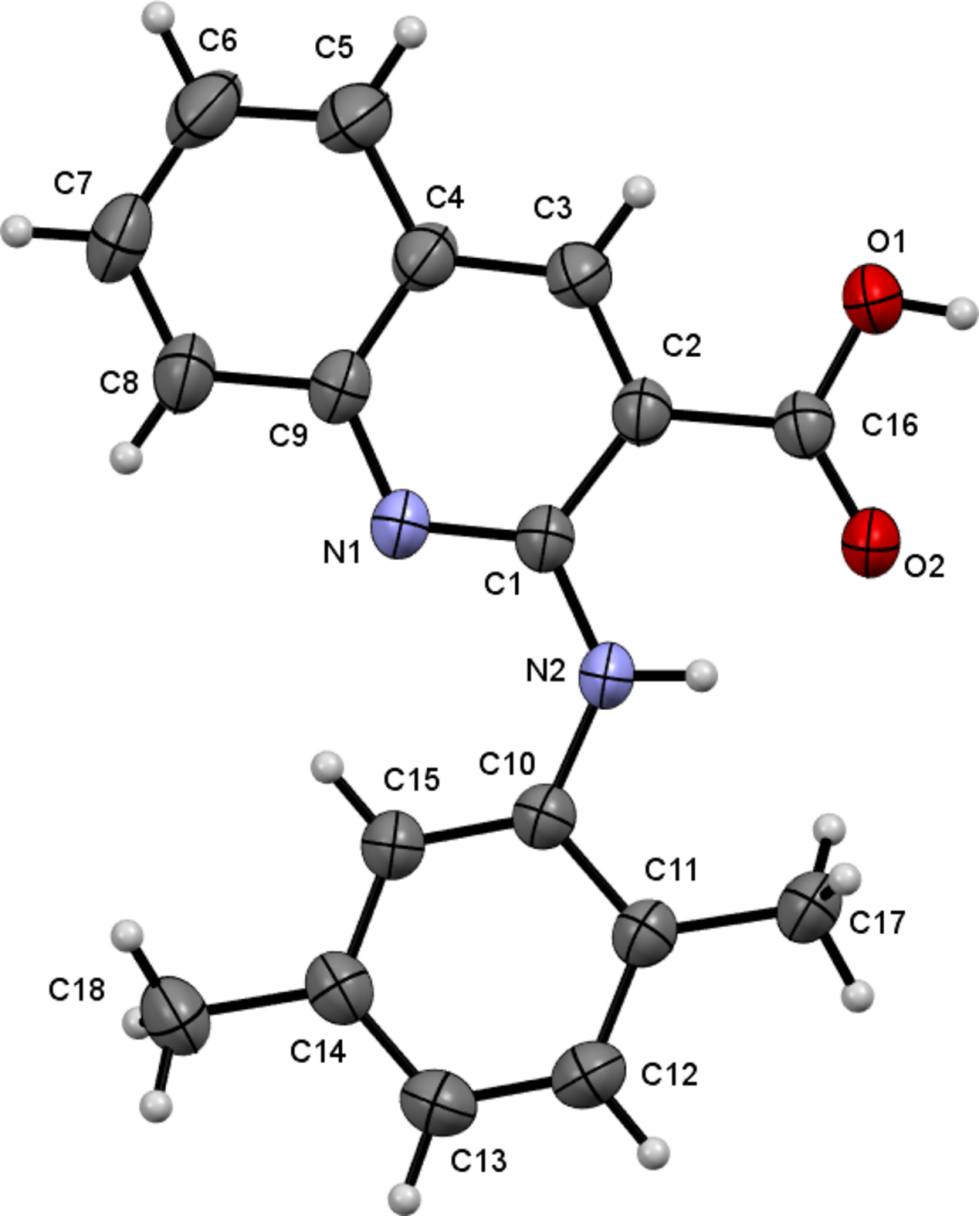
The mol­ecular structure of the title compound with displacement ellipsoids drawn at the 50% probability level.

**Figure 2:**
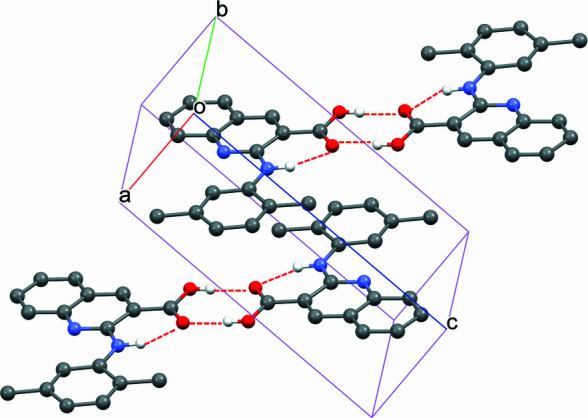
Packing of the mol­ecules in the title compound (for clarity, H atoms not involved in hydrogen bonding are omitted).

In the title compound (Fig. 1 ▸), the two aromatic moieties are nearly coplanar with a dihedral angle of 6.51 (5)°. In the crystal (Fig. 2 ▸), adjacent mol­ecules form carb­oxy­lic acid dimers *via* inter­molecular hydrogen bonding (Table 1 ▸).

## Synthesis and crystallization

**Figure 3:**
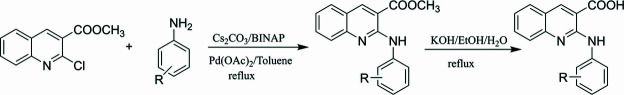
Synthesis of the title compound.

The target compound 2-[(2,5-di­methyl­phen­yl)amino]­quinoline-3-carb­oxy­lic acid was synthesized (Fig. 3 ▸) *via* a two-step route with the Buchwald–Hartwig cross-coupling reaction. In the first step, methyl 2-chloro­quinoline-3-carboxyl­ate reacted with 2,5-di­methyl­aniline in toluene for 24 h using Pd(OAc)_2_/BINAP as the catalytic system and Cs_2_CO_3_ as the base. The inter­mediate methyl 2-[(2,5-di­methyl­phen­yl)amino]­quinoline-3-carboxyl­ate was obtained by extraction followed by column chromatography. In the second step, the above inter­mediate was hydrolyzed in an aqueous ethanol solution containing KOH for 6 h. After the reaction, the mixture was acidified, and the target product was isolated by extraction and purified by column chromatography. Pure 2-[(2,5-di­methyl­phen­yl)amino]­quinoline-3-carb­oxy­lic acid was dried for 8 h. Single crystals were obtained by slow evaporation of an ethanol solution at room temperature.

## Refinement

Crystal data, data collection and structure refinement details are summarized in Table 2 ▸.

## Supplementary Material

Crystal structure: contains datablock(s) global, I. DOI: 10.1107/S2414314626005468/bx4040sup1.cif

Structure factors: contains datablock(s) I. DOI: 10.1107/S2414314626005468/bx4040Isup2.hkl

CCDC reference: 2556276

Additional supporting information:  crystallographic information; 3D view; checkCIF report

## Figures and Tables

**Table 1:** Hydrogen-bond geometry (Å, °)

*D*—H⋯*A*	*D*—H	H⋯*A*	*D*⋯*A*	*D*—H⋯*A*
N2—H2⋯O2	0.86	1.96	2.6925 (13)	142
C15—H15⋯N1	0.93	2.30	2.9134 (15)	123
O1—H1⋯O2^i^	0.82	1.85	2.6702 (12)	178

Symmetry code: (i) 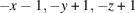.

**Table 2:** Experimental details

Crystal data	
Chemical formula	C_18_H_16_N_2_O_2_
*M*_r_	292.33
Crystal system, space group	Triclinic, *P* 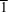
Temperature (K)	299
*a*, *b*, *c* (Å)	4.80942 (7), 11.8944 (2), 12.9466 (2)
α, β, γ (°)	88.9803 (13), 85.8751 (12), 84.4910 (12)
*V* (Å^3^)	735.24 (2)
*Z*	2
Radiation type	Cu *K*α
μ (mm^−1^)	0.70
Crystal size (mm)	0.21 × 0.05 × 0.04

Data collection	
Diffractometer	XtaLAB Synergy R, DW system, HyPix
Absorption correction	Multi-scan (*CrysAlis PRO*; Rigaku OD, 2024 ▸)
*T*_min_, *T*_max_	0.805, 1.000
No. of measured, independent and observed [*I* > 2σ(*I*)] reflections	7863, 2904, 2659
*R*_int_	0.021
(sin θ/λ)_max_ (Å^−1^)	0.629

Refinement	
*R*[*F*^2^ > 2σ(*F*^2^)], *wR*(*F*^2^), *S*	0.039, 0.118, 1.07
No. of reflections	2904
No. of parameters	203
H-atom treatment	H-atom parameters constrained
Δρ_max_, Δρ_min_ (e Å^−3^)	0.22, −0.16

Computer programs: *CrysAlis PRO* (Rigaku OD, 2024 ▸), *SHELXT* (Sheldrick, 2015*a* ▸), *SHELXL2018/3* (Sheldrick, 2015*b* ▸), *OLEX2* (Dolomanov *et al.*, 2009 ▸) and *Mercury* (Macrae *et al.*, 2020 ▸).

## full crystallographic data

### 2-[(2,5-Dimethylphenyl)amino]quinoline-3-carboxylic acid Crystal data

**Table:**

C_18_H_16_N_2_O_2_	*Z* = 2
*M**_r_* = 292.33	*F*(000) = 308
Triclinic, *P*1	*D*_x_ = 1.320 Mg m^−^^3^
*a* = 4.80942 (7) Å	Cu *K*α radiation, λ = 1.54184 Å
*b* = 11.8944 (2) Å	Cell parameters from 2760 reflections
*c* = 12.9466 (2) Å	θ = 3.4–74.8°
α = 88.9803 (13)°	µ = 0.70 mm^−^^1^
β = 85.8751 (12)°	*T* = 299 K
γ = 84.4910 (12)°	Needle, clear dark yellow
*V* = 735.24 (2) Å^3^	0.21 × 0.05 × 0.04 mm

### 2-[(2,5-Dimethylphenyl)amino]quinoline-3-carboxylic acid Data collection

**Table:**

XtaLAB Synergy R, DW system, HyPix diffractometer	2904 independent reflections
Radiation source: Rotating-anode X-ray tube, Rigaku (Cu) X-ray Source	2659 reflections with *I* > 2σ(*I*)
Mirror monochromator	*R*_int_ = 0.021
Detector resolution: 10.0000 pixels mm^-1^	θ_max_ = 76.0°, θ_min_ = 3.4°
ω scans	*h* = −5→5
Absorption correction: multi-scan (CrysAlisPro; Rigaku OD, 2024)	*k* = −14→14
*T*_min_ = 0.805, *T*_max_ = 1.000	*l* = −16→16
7863 measured reflections	

### 2-[(2,5-Dimethylphenyl)amino]quinoline-3-carboxylic acid Refinement

**Table:**

Refinement on *F*^2^	Hydrogen site location: inferred from neighbouring sites
Least-squares matrix: full	H-atom parameters constrained
*R*[*F*^2^ > 2σ(*F*^2^)] = 0.039	*w* = 1/[σ^2^(*F*_o_^2^) + (0.0644*P*)^2^ + 0.1121*P*] where *P* = (*F*_o_^2^ + 2*F*_c_^2^)/3
*wR*(*F*^2^) = 0.118	(Δ/σ)_max_ < 0.001
*S* = 1.07	Δρ_max_ = 0.22 e Å^−^^3^
2904 reflections	Δρ_min_ = −0.16 e Å^−^^3^
203 parameters	Extinction correction: SHELXL2018/3 (Sheldrick 2015b), Fc^*^=kFc[1+0.001xFc^2^λ^3^/sin(2θ)]^-1/4^
0 restraints	Extinction coefficient: 0.0035 (12)
Primary atom site location: dual	

### 2-[(2,5-Dimethylphenyl)amino]quinoline-3-carboxylic acid Special details

**Table:**

Geometry. All esds (except the esd in the dihedral angle between two l.s. planes) are estimated using the full covariance matrix. The cell esds are taken into account individually in the estimation of esds in distances, angles and torsion angles; correlations between esds in cell parameters are only used when they are defined by crystal symmetry. An approximate (isotropic) treatment of cell esds is used for estimating esds involving l.s. planes.
Refinement. The position of the H atom in O and the position of the H atom in C are obtained from the differential Fourier diagram. The geometric positioning of the H atom is C—H = 0.93 for the aromatic group and the geometric positioning of the H atom is O—H = 0.82 for the methyl group.

### 2-[(2,5-Dimethylphenyl)amino]quinoline-3-carboxylic acid Fractional atomic coordinates and isotropic or equivalent isotropic displacement parameters (Å^2^)

**Table:**

	*x*	*y*	*z*	*U*_iso_*/*U*_eq_	
O1	−0.35770 (17)	0.61275 (7)	0.41684 (7)	0.0472 (2)	
H1	−0.479882	0.599790	0.461771	0.071*	
O2	−0.23465 (17)	0.42979 (7)	0.44090 (6)	0.0456 (2)	
N1	0.4312 (2)	0.45264 (8)	0.20624 (7)	0.0415 (3)	
N2	0.2069 (2)	0.33488 (8)	0.32298 (8)	0.0437 (3)	
H2	0.070482	0.332918	0.369511	0.052*	
C1	0.2313 (2)	0.43900 (9)	0.27944 (8)	0.0360 (3)	
C2	0.0353 (2)	0.53280 (9)	0.31657 (8)	0.0362 (3)	
C3	0.0700 (2)	0.63768 (10)	0.27559 (9)	0.0429 (3)	
H3	−0.050859	0.699194	0.298862	0.052*	
C4	0.2853 (2)	0.65434 (10)	0.19880 (9)	0.0428 (3)	
C5	0.3314 (3)	0.76093 (12)	0.15430 (12)	0.0604 (4)	
H5	0.221114	0.825172	0.177780	0.072*	
C6	0.5367 (3)	0.77007 (13)	0.07711 (12)	0.0641 (4)	
H6	0.565416	0.840374	0.047888	0.077*	
C7	0.7041 (3)	0.67368 (13)	0.04186 (11)	0.0585 (4)	
H7	0.841604	0.680419	−0.011595	0.070*	
C8	0.6686 (3)	0.57010 (12)	0.08475 (10)	0.0510 (3)	
H8	0.783743	0.507189	0.061076	0.061*	
C9	0.4577 (2)	0.55755 (10)	0.16501 (9)	0.0396 (3)	
C10	0.3639 (2)	0.23006 (9)	0.30574 (9)	0.0392 (3)	
C11	0.2930 (2)	0.14151 (10)	0.37352 (9)	0.0426 (3)	
C12	0.4354 (3)	0.03583 (11)	0.35730 (11)	0.0518 (3)	
H12	0.390172	−0.023759	0.400831	0.062*	
C13	0.6432 (3)	0.01616 (11)	0.27818 (11)	0.0532 (3)	
H13	0.733864	−0.055992	0.269038	0.064*	
C14	0.7167 (3)	0.10343 (11)	0.21262 (10)	0.0459 (3)	
C15	0.5758 (2)	0.21017 (10)	0.22698 (9)	0.0436 (3)	
H15	0.623613	0.269343	0.183389	0.052*	
C16	−0.1953 (2)	0.51938 (9)	0.39614 (8)	0.0365 (3)	
C17	0.0690 (3)	0.15969 (11)	0.46105 (10)	0.0519 (3)	
H17A	0.101986	0.224403	0.500077	0.078*	
H17B	0.073923	0.094170	0.505505	0.078*	
H17C	−0.111249	0.172065	0.433234	0.078*	
C18	0.9457 (3)	0.08438 (12)	0.12718 (11)	0.0586 (4)	
H18A	0.919523	0.141436	0.074471	0.088*	
H18B	0.938963	0.011242	0.097787	0.088*	
H18C	1.124263	0.088331	0.154902	0.088*	

### 2-[(2,5-Dimethylphenyl)amino]quinoline-3-carboxylic acid Atomic displacement parameters (Å^2^)

**Table:**

	*U*^11^	*U*^22^	*U*^33^	*U*^12^	*U*^13^	*U*^23^
O1	0.0412 (5)	0.0431 (5)	0.0534 (5)	0.0014 (3)	0.0146 (4)	0.0031 (4)
O2	0.0426 (4)	0.0430 (5)	0.0478 (5)	−0.0009 (3)	0.0145 (3)	0.0055 (4)
N1	0.0430 (5)	0.0402 (5)	0.0397 (5)	−0.0053 (4)	0.0101 (4)	0.0021 (4)
N2	0.0438 (5)	0.0379 (5)	0.0460 (5)	−0.0023 (4)	0.0172 (4)	0.0046 (4)
C1	0.0355 (5)	0.0381 (6)	0.0339 (5)	−0.0050 (4)	0.0038 (4)	0.0008 (4)
C2	0.0330 (5)	0.0404 (6)	0.0346 (5)	−0.0040 (4)	0.0019 (4)	0.0015 (4)
C3	0.0399 (6)	0.0404 (6)	0.0466 (6)	0.0001 (4)	0.0037 (5)	0.0042 (5)
C4	0.0410 (6)	0.0431 (6)	0.0440 (6)	−0.0059 (5)	0.0008 (5)	0.0081 (5)
C5	0.0599 (8)	0.0465 (7)	0.0715 (9)	−0.0028 (6)	0.0101 (7)	0.0167 (7)
C6	0.0665 (9)	0.0556 (8)	0.0692 (9)	−0.0152 (7)	0.0087 (7)	0.0243 (7)
C7	0.0580 (8)	0.0679 (9)	0.0493 (7)	−0.0190 (7)	0.0125 (6)	0.0115 (6)
C8	0.0519 (7)	0.0553 (7)	0.0447 (7)	−0.0115 (6)	0.0126 (5)	0.0025 (6)
C9	0.0392 (6)	0.0455 (6)	0.0345 (5)	−0.0091 (5)	0.0012 (4)	0.0031 (5)
C10	0.0391 (6)	0.0359 (6)	0.0418 (6)	−0.0045 (4)	0.0041 (4)	0.0000 (4)
C11	0.0425 (6)	0.0401 (6)	0.0449 (6)	−0.0081 (5)	0.0051 (5)	0.0009 (5)
C12	0.0571 (7)	0.0380 (6)	0.0587 (8)	−0.0062 (5)	0.0067 (6)	0.0071 (5)
C13	0.0554 (7)	0.0382 (6)	0.0630 (8)	0.0034 (5)	0.0068 (6)	−0.0009 (6)
C14	0.0445 (6)	0.0455 (6)	0.0459 (6)	−0.0003 (5)	0.0048 (5)	−0.0044 (5)
C15	0.0450 (6)	0.0400 (6)	0.0439 (6)	−0.0029 (5)	0.0084 (5)	0.0018 (5)
C16	0.0327 (5)	0.0405 (6)	0.0357 (5)	−0.0021 (4)	0.0006 (4)	−0.0004 (4)
C17	0.0580 (8)	0.0431 (7)	0.0526 (7)	−0.0102 (5)	0.0160 (6)	0.0049 (5)
C18	0.0585 (8)	0.0562 (8)	0.0560 (8)	0.0074 (6)	0.0148 (6)	−0.0047 (6)

### 2-[(2,5-Dimethylphenyl)amino]quinoline-3-carboxylic acid Geometric parameters (Å, º)

**Table:**

O1—C16	1.3150 (13)	C5—C6	1.364 (2)
O2—C16	1.2281 (13)	C6—C7	1.400 (2)
N1—C1	1.3199 (14)	C7—C8	1.3631 (19)
N1—C9	1.3621 (15)	C8—C9	1.4149 (15)
N2—C1	1.3633 (14)	C10—C11	1.4102 (16)
N2—C10	1.4070 (14)	C10—C15	1.3954 (15)
C1—C2	1.4549 (15)	C11—C12	1.3844 (18)
C2—C3	1.3672 (16)	C11—C17	1.5099 (16)
C2—C16	1.4765 (14)	C12—C13	1.3855 (19)
C3—C4	1.4087 (16)	C13—C14	1.3852 (18)
C4—C5	1.4135 (17)	C14—C15	1.3894 (17)
C4—C9	1.4095 (17)	C14—C18	1.5078 (17)
			
C1—N1—C9	119.30 (10)	N1—C9—C8	118.53 (11)
C1—N2—C10	131.81 (9)	C4—C9—C8	118.43 (11)
N1—C1—N2	119.81 (10)	N2—C10—C11	115.75 (10)
N1—C1—C2	121.83 (10)	C15—C10—N2	124.25 (10)
N2—C1—C2	118.36 (9)	C15—C10—C11	120.00 (11)
C1—C2—C16	122.98 (10)	C10—C11—C17	121.81 (11)
C3—C2—C1	117.75 (10)	C12—C11—C10	117.80 (11)
C3—C2—C16	119.27 (10)	C12—C11—C17	120.38 (11)
C2—C3—C4	121.26 (11)	C11—C12—C13	121.98 (11)
C3—C4—C5	123.55 (12)	C14—C13—C12	120.32 (11)
C3—C4—C9	116.75 (11)	C13—C14—C15	118.79 (11)
C9—C4—C5	119.68 (11)	C13—C14—C18	121.21 (11)
C6—C5—C4	120.36 (14)	C15—C14—C18	120.00 (11)
C5—C6—C7	120.03 (12)	C14—C15—C10	121.09 (11)
C8—C7—C6	120.95 (12)	O1—C16—C2	114.30 (9)
C7—C8—C9	120.51 (13)	O2—C16—O1	121.77 (9)
N1—C9—C4	123.04 (10)	O2—C16—C2	123.92 (10)

### 2-[(2,5-Dimethylphenyl)amino]quinoline-3-carboxylic acid Hydrogen-bond geometry (Å, º)

**Table:**

*D*—H···*A*	*D*—H	H···*A*	*D*···*A*	*D*—H···*A*
N2—H2···O2	0.86	1.96	2.6925 (13)	142
C15—H15···N1	0.93	2.30	2.9134 (15)	123
O1—H1···O2^i^	0.82	1.85	2.6702 (12)	178

Symmetry code: (i) −*x*−1, −*y*+1, −*z*+1.
